# Cancer-Associated Fibroblasts: Master Tumor Microenvironment Modifiers

**DOI:** 10.3390/cancers15061899

**Published:** 2023-03-22

**Authors:** Kellen Wright, Thuc Ly, Matthew Kriet, Andras Czirok, Sufi Mary Thomas

**Affiliations:** 1 Department of Otolaryngology, University of Kansas Medical Center, Kansas City, KS 66160, USA; 2 Department of Cancer Biology, University of Kansas Medical Center, Kansas City, KS 66160, USA; 3 Department of Cell Biology and Physiology, University of Kansas Medical Center, Kansas City, KS 66160, USA

**Keywords:** extracellular matrix, tumor microenvironment, cancer-associated fibroblasts, metastasis

## Abstract

**Simple Summary:**

Cancer cells rely on the surrounding environment to grow and spread. This environment contains structural components, secreted factors, immune cells, and other types of cells. Among these, cancer-associated fibroblasts (CAFs) play an important role in tumor cell growth, invasion, and metastasis. CAFs are heterogenous within tumor tissue and across cancer types. Given the plasticity, CAFs are the master tumor microenvironment modifier: an architect and a coordinator. Specifically, they produce and remodel the tumor microenvironment. They also communicate with immune cells, aiding the cancer in evading immune detection. This review highlights the factors and signaling pathways by which CAFs act.

**Abstract:**

Cancer cells rely on the tumor microenvironment (TME), a composite of non-malignant cells, and extracellular matrix (ECM), for survival, growth, and metastasis. The ECM contributes to the biomechanical properties of the surrounding tissue, in addition to providing signals for tissue development. Cancer-associated fibroblasts (CAFs) are stromal cells in the TME that are integral to cancer progression. Subtypes of CAFs across a variety of cancers have been revealed, and each play a different role in cancer progression or suppression. CAFs secrete signaling molecules and remodel the surrounding ECM by depositing its constituents as well as degrading enzymes. In cancer, a remodeled ECM can lead to tumor-promoting effects. Not only does the remodeled ECM promote growth and allow for easier metastasis, but it can also modulate the immune system. A better understanding of how CAFs remodel the ECM will likely yield novel therapeutic targets. In this review, we summarize the key factors secreted by CAFs that facilitate tumor progression, ECM remodeling, and immune suppression.

## 1. Introduction

Cancer research for many years has focused on molecular perturbations in the tumor cells that contribute to progression and metastasis. The microenvironment surrounding the tumor cells plays an important role in progression. The tumor microenvironment (TME) is made up of non-cellular components including the extracellular matrix (ECM) and stromal cells. One of the most prominent cells in the TME are cancer-associated fibroblasts (CAFs), which can either impair or facilitate tumor metastasis [1].

Historically discovered as a major component in connective tissues with a distinguished morphology, as elongated, spindle-like cells, fibroblasts are easily propagated in vitro [2]. Fibroblasts are normally quiescent; upon tissue injury, fibroblasts are activated to become myofibroblasts that proliferate and secrete growth factors, cytokines, and ECM proteins for wound healing [3]. Injured tissues release factors (e.g., TGF-β, PDGF, HGF, FGF2, inflammatory cytokines, metalloproteinases, and reactive oxygen species) that activate fibroblasts to acquire characteristics of smooth muscle cells, including the expression of α-smooth muscle actin (α-SMA) and contractile abilities [1,4,5]. As tumors resemble “wounds that do not heal”, they induce CAFs that form desmoplastic lesions of tumors, with α-SMA as one of the earliest described markers for CAFs [6]. Along with the advancement of molecular techniques, other markers of CAFs that correlate with poor patient survival (implicating the “protumor” perspective at a time) such as FSP1, FAP, vimentin, PDGFR-a, PDGFR-b, NG2, and CXCL12 have been identified [7,8]. However, none of these “markers” are exclusive to CAFs. In practice, CAFs are identified based on a combination of morphology, spindle shape, tissue position, and negative expression for epithelial cells (EpCAM), endothelial cells (CD31), immune cells (CD45), and pericytes (NG2) [9]. With the advancement of flow cytometry and single-cell RNA sequencing, it is evident that CAFs within tumor tissues are heterogenous in the transcriptional profile, and they are diverse across the cancer type as well. For example, there are four CAF subpopulations in breast cancer [10], three in PDAC [11,12], three in HNSCC [13], four in lung cancer [14], two in colorectal cancer [15], and three in glioblastoma [16]. The role of CAFs in tumor progression is complex and nuanced beyond the initial protumor effects reported. Crosstalk with other stromal cell types in the tumor tissue through secreted metabolites, cytokines, and signaling molecules makes CAFs important regulators of the TME. In this review, we aim to provide an overview of CAF effects upon the TME. We will also capture the essence of CAFs: as architects of the ECM and angiogenesis.

## 2. CAFs Modulate the ECM Composition

The ECM serves as a scaffold for cells while regulating cellular activity and gene expression. The ECM is a composite of various molecules, such as proteins (e.g., collagens, elastin, proteoglycans, and glycoproteins), which include laminins and fibronectin. Each component can be detected by specialized cell-surface receptors, which collectively determine the material and transport properties of the tissue microenvironment. The composition of the ECM is tissue-, organ-, and species-specific [17]. The most basic classification of ECM types distinguishes the interstitial matrix from the basement membrane. The basement membrane is a denser collection of collagen IV and laminins that typically separate the epithelium from the underlying connective tissue, e.g., the epidermal layer from the dermis. The interstitial matrix comprises the three-dimensional mesh structure that typically consists of collagens I, III, and V, elastin, and fibronectin [18,19].

The ECM is a dynamic structure undergoing constant remodeling. Fibroblasts most commonly synthesize and secrete components of the ECM as well as extracellular enzymes that degrade portions of the ECM. In homeostasis, ECM production and degradation are in a balance that is responsive to changes in tissue function, including mechanical load bearing. Matrix metalloproteinases (MMPs) are the primary enzymes responsible for ECM degradation [20]. The activity of ECM-degrading enzymes is controlled by complex biological pathways, with tissue inhibitors of metalloproteinases (TIMPs) playing an important role [21]. Other enzymes are responsible for remodeling ECM, which includes plasmin, cathepsins, heparanases, and sulfatases [22].

ECM stiffness, a major marker of cancer’s progressive and invasive potentials, is affected by the composition of the ECM [23]. The stiffness of the matrix impacts cancer cell proliferation, and aids in the resistance to treatment [24]. The yes-associated protein (YAP) and transcriptional coactivator with a PDZ-binding motif (TAZ) have been implicated in CAFs and poor prognosis in cancer. YAP/TAZ are oncoproteins that are active through the Hippo pathway [25]. Increased YAP/TAZ signaling results in the remodeling of the extracellular matrix and cell differentiation [26]. Additionally, the regulation of this pathway is largely controlled by physical cues in the surrounding microenvironment. Specifically, matrix stiffness has been shown to modulate the YAP/TAZ pathway. One mediator of YAP/TAZ remodeling, RAP2, is activated when ECM stiffness is low, inhibiting YAP/TAZ [27]. Calvo et al. demonstrated the activation of the YAP/TAZ pathway in CAFs cocultured with breast cancer cell lines [28]. YAP/TAZ-induced cytoskeletal regulators, including the actin-binding protein anillin (ANLN) and the actin-elongation regulator Diaphanous-Related Formin 3 (DIAPH3), contribute to matrix stiffening. Perturbation of YAP/TAZ rendered CAFs that were much less pro-tumorigenic. A positive feedback loop through activation of the YAP/TAZ pathway and increased matrix stiffness exacerbated the matrix stiffening. In another study, glycoprotein dickkopf-3 (DKK3) was identified as a mediator of CAF-regulated matrix stiffening. DKK3 acts by increasing the YAP/TAZ and Wnt/β-catenin signaling pathways [29].

Collagens, which are the most abundant protein family in the human body, are deposited by fibroblasts and many other types of cells. The structure, stiffness, motility, and signaling potential of the ECM are all influenced by the balance of collagen fibers that make up the ECM [30]. Fibrillar collagen I, the most abundant collagen, is a fibril-forming collagen that is often secreted by fibroblasts. Collagen crosslinking is an essential part of ECM stiffening. The lysyl oxidase (LOX) enzyme is responsible for the crosslinking of collagen I. The inhibition of LOX resulted in decreased stiffness and increased tumor latency [31]. In cancer types with increased CAF-expressed LOX, the ECM collagen I levels are higher. Breast cancer with CAFs expressing LOX has a higher rate of invasion and metastasis [32,33]. Similar observations were noted in ovarian, lung, HNSCC, esophageal adenocarcinoma, and colorectal cancers. An increased collagen fiber length was linked to a poor prognosis [34,35]. Li et al. demonstrated that CAF-secreted LOX at the metastatic niche is associated with a worse prognosis in gastric cancer patients [36]. Further, TGF-β signaling in CAFs induced the expression of LOX. The LOX inhibitor BAPN the reduced metastasis of gastric cancer to the liver.

ECM components may facilitate tumor suppression in some cases and can be tumor-promoting in others. For example, Brisson et al. demonstrated that murine mammary carcinoma was more aggressive and metastasized more often in the collagen III-deficient murine mammary cell line 4T1 [37,38]. However, collagen III was reported to facilitate cancer cell migration in prostate cancer [39]. The depletion of type V collagen reduced tumor growth in mammary carcinoma [40].

Type VI collagen is found to be upregulated specifically in CAFs in colorectal carcinoma (CRC) [41]. Other cancers that were reported to have highly expressed type VI collagen include melanoma, ovarian cancer, lung cancer, esophageal cancer, pancreatic cancer, breast cancer, and more. Col6a1^−/−^ mice with B16F10 melanoma allografts in the brain had decreased tumor proliferation compared to wild-type control mice due to reduced vasculature. Other effects of type VI collagen include the recruitment of macrophages, increases in inflammation, and angiogenesis [42].

In HNSCC, collagen XI α1 (colXIα1) was elevated in comparison to controls in tumor cells and stromal fibroblasts, and the knockdown of colXIα1 restricted HNSCC cell proliferation in vitro. This illustrates a pro-tumor effect of type XI collagen [43,44]. Less is known about collagen types XXII and XXIV; however, increased mRNA expression in HNSCC is associated with metastasis and recurrence [45].

Tissue inhibitors of matrix metalloproteinases (TIMPS) are multi-faceted proteins that act downstream of multiple signaling pathways to inhibit MMPs [46]. TIMPS have been implicated in cancer progression and metastasis. For instance, CAF-secreted TIMP-1 initiates a STAT3 feedback loop that has been shown to play an integral part in breast cancer growth and migration [47].

MMPs are important players in ECM dynamics. MMP-2 is elevated in both CAFs and bladder cancer cell lines, which contributes to tumor growth [48]. Further, CAFs induce MMP-2 and -14 expression in cancer cells [49]. In gastric cancer, CAF-mediated signaling induces the expression of MMP-2/9, and TIMP-1/2 increases the tumor invasion by activating the JAK2/STAT3 pathway [50].

Fibronectin is a component of the ECM that is deposited by fibroblasts. In cancer, fibronectin is implicated in the invasion. Fibronectin is secreted by CAFs that are isolated from colon cancer patients at a higher rate than normal fibroblasts [51]. The depletion of fibronectin in CAFs through siRNA silencing reduced the cancer’s ability to invade, indicating that fibronectin is required for CAF-mediated tumor invasiveness. However, CAFs that are lacking in fibronectin retained their ability to apply mechanical force and organize the collagen network. Glentis et al. demonstrated that physical contact between CAFs and tumor cells was required to assist with invasion, and that this process was independent to MMP activity [52]. Using live imaging of CAFs moving along the basement membrane, they showed that CAFs widened holes in the basement membrane, thus allowing cancer cells to penetrate and invade.

In addition to modifying the entire surrounding tumor stroma, CAFs can remodel specific parts of the ECM, allowing for easier invasion. Reported by Gaggioli et al., CAFs lead invading cancer cells, leaving an invasion-friendly pathway in their wake [53]. These “tracks,” provide a permissive environment that allows for a more frequent and aggressive invasion. The tracks are characterized by “thick collagen bundles around their sides and an absence of matrix in their center,” and the deposition of fibronectin and tenascin-C. The presence of tumor cells is not required for track formation. Track formation is partly mediated by the Rho/ROCK pathway in CAFs, and inhibition of these pathways at several steps limits the amount of remodeling that is performed.

Using a simulated cancer environment, experiments showed that the migration of fibroblasts creates a permissive environment for tumor cells to follow. The migration of fibroblasts toward lymphatics is linked to an increasing concentration of TGF-β in the direction of movement. Tumor cells can also physically attach themselves to moving CAFs, allowing for the movement of tumor cells. This increased velocity leads to greater tumor aggressiveness [54]. Podoplanin, a glycoprotein, modulates track formation and invasiveness. Cancers with high podoplanin-positive CAF populations correlate with poor clinical outcomes. CAFs that express podoplanin also further invade the ECM in both in vitro and in vivo studies. Podoplanin acts via the Rho/ROCK signaling pathway [55]. In CAFs, the Rho/ROCK pathway modulates actomyosin contractility and is shown to be a requirement for force-mediated ECM remodeling [56].

Discoidin domain receptor 2 (DDR2), a fibrillar collagen receptor, is present on both tumor cells and CAFs [57]. Increased expression is related to tumor metastasis in breast cancer in mice, without affecting primary tumor growth. In CAFs, DDR2 expression is directly related to the ability of CAFs to remodel the ECM. The depletion of DDR2 in CAFs changed the resulting ECM to resemble an ECM produced by normal fibroblasts.

While the effect of CAFs on the ECM and surrounding cells is typically tumor-promoting, the heterogeneity of CAF populations allows for different pro-tumor effects, with some populations reported to restrict tumor growth [8]. αSMA+ fibroblasts, also known as myCAFs in pancreatic cancer, have been recently shown to slow tumor progression in colorectal cancer. Reduction of αSMA+ CAFs in mouse models fostered a more immunosuppressive TME [58]. Other experiments have eliminated αSMA+, potentially targeting tumor-restraining CAFs and not tumor-promoting CAFs [59]. αSMA is not uniformly positive in all CAFs, and it has varying expression levels within αSMA+ CAFs [8]. Meflin, a protein present in some CAF populations, is essential for anti-tumor effects. The reduction of meflin in these populations resulted in a more aggressive pancreatic ductal adenocarcinoma (PDAC) course in mouse models [60]. The cancer-restraining properties of CAFs are not well understood. Future research into pro- and anti-tumor properties and the heterogeneity of CAFs will allow an improved method of targeting the TME in cancer treatment.

## 3. CAF-Secreted Factors Facilitate Tumor Progression

CAFs adopt an activated phenotype that secretes several factors that facilitate tumor growth and metastasis. These factors can act in a paracrine manner: they bind to the cell surface receptors of nearby cells and trigger downstream molecular signaling pathways (Table 1).

**Figure 1:**
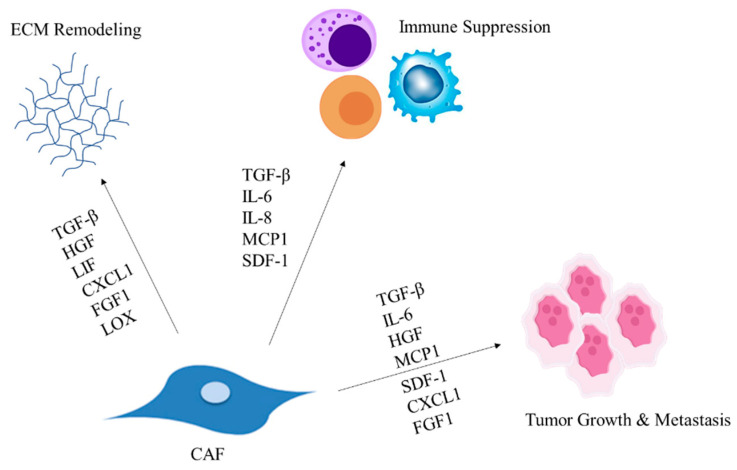
CAF factors influence pro-tumor processes. CAF-derived molecules TGF-β, HGF, LIF, CXCL1, and FGF1 impact ECM remodeling. Factors SDF-1, MCP1, IL-8, Il-8, and TGF-β modulate the immune system. TGF-β, IL-6, HGF, MCP1, SDF-1, CXCL1, and FGF1 impact tumor growth and metastasis.

Both tumor and stromal cells in the microenvironment respond to CAF-secreted factors (Figure 1). Transforming growth factor-β (TGF-β), has wide-ranging effects on physiological processes as well as cancer [85]. It contributes toward tumor epithelial to mesenchymal transition (EMT) and invasiveness [65]. This cytokine is implicated in driving the formation of LRRC15+ CAFs [86]. Activated CAFs secrete TGF-β, leading to a positive feedback loop prompting the secretion of lysyl oxidase (LOX) enzymes from CAFs and other cells. Specifically, the secretion of LOX like-2, promotes collagen crosslinking, increasing the stiffness of the ECM in hepatocellular carcinoma (HCC) [64]. TGF-β also increases fibronectin synthesis, thus promoting a matrix-associated fibronectin assembly process called fibrillogenesis [63]. In addition, it also activates immune cells such as M2 macrophages, thus producing inflammation and the deposition of ECM components in the liver resulting in fibrosis [61]. In mammary tumors, autocrine signaling of TGF-β in fibroblasts sustains the CAF phenotype. In addition, TGF-β along with stromal cell-derived factor 1 (SDF-1) facilitate the development of tumor-promoting CAFs [62].

In CRC, CAFs have been reported to secrete IL-6, which is a cytokine with pleiotropic activity including inflammation and angiogenesis. Tumor-derived IL-6 induces the vascular endothelial growth factor (VEGF) release from CAFs [69]. In pancreatic cancer, increased IL-6 expression was associated with an increased immunosuppressive stroma. A reduction in the amount of collagen in the ECM was noted with an interleukin 6 receptor (IL6R) antibody treatment [68]. IL-6 is shown to act through the STAT3 pathway [87]. Head and neck squamous cell carcinoma (HNSCC) tumors have an intricate mechanism of inducing a process called secretory autophagy in CAFs [88]. HNSCC-secreted FGF2 induces the STAT3-mediated transcription of SOX2, which in turn suppresses the expression of mTOR inducing secretory autophagy in CAFs. Several factors including IL-6 and IL-8 are secreted from CAFs into the tumor microenvironment.

The hepatocyte growth factor (HGF), also known as scatter factor, is secreted by CAFs in some cancers [89]. HGF contributes to desmoplasia as well as to the acidification of the TME through the regulation of tumor metabolism. In ovarian cancer cells, HGF secreted by CAFs increased the therapeutic resistance and cell proliferation [75]. In HNSCC, HGF induces glycolysis, which facilitates the secretion of tumor-derived lactate [4]. CAFs in turn, respond to HNSCC-released FGF2 to increase lactate uptake and mitochondrial oxidative phosphorylation. Inhibiting the HGF/c-Met pathway reduced HNSCC tumor growth [4,90].

The leukemia inhibitory factor (LIF) is another signaling molecule that is implicated in the remodeling of extracellular matrix. The tumor secreted LIF promotes CAF activation and the expression of LIF in fibroblasts [77]. Further, LIF regulates pro-invasive effects of CAFs on HNSCC through the regulation of actomyosin contractility, which is independent of α-SMA expression in CAFs. LIF also facilitates the pro-tumor effects of TGF-β and is correlated with a poor clinical prognosis.

The stromal cell-derived factor 1 (SDF-1), also known as C-X-C motif chemokine 12 (CXCL12), can facilitate macrophage recruitment during cancer progression. Inhibiting SDF-1 restricted macrophage migration at an even higher rate than MCP-1 inhibition in CAFs associated with breast cancer cells [72]. SDF-1 expression is a poor prognostic indicator in endometrial cancer. Working through the SDF-1/CXCR4 pathway, SDF-1 increased MMP-2/MMP-9 production and led to an increased tumor invasiveness [91]. Zhu et al. further investigated the pathways involving CXCL12 [92]. TWIST1 is a transcription factor that when overexpressed, leads to increased amounts of CXCL12. The knockdown of TWIST1 in CAFs limited the epithelial–mesenchymal transition (EMT) of esophageal cancer cells.

C-X-C motif chemokine ligand 1 (CXCL1) is highly expressed in HNSCC CAFs and is associated with poor survival. Further, CAF-conditioned media or CAF-secreted CXCL1 increased the tumor invasion [93]. This expanded invasive capacity is a result of the CXCL1-mediated increase in CAF-secreted MMP1.

In experiments with CRC, FGF1 expression by CAFs was increased alongside the activation of its receptor, FGFR3. When FGF1 expression was reduced, the migratory ability of the tumor cells was reduced. These FGF1-producing fibroblasts were determined to be FAP-positive CAFs [83]. In lung cancer, CAFs were shown to overexpress FGF2 and FGF9. These CAF populations secreted high levels of collagen and contributed to tumor aggressiveness [82]. FGF2 signaling activated pathways that led to the acquisition of a CAF phenotype [94].

Du et al. demonstrated that Hic-5, an intracellular protein expressed at higher levels in CAFs than normal fibroblasts, is associated with an increased lymph node metastasis. Specifically, experiments indicated that Hic-5 enhanced cell migration and invasion in esophageal cancer. Stromal components such as MMP-2 were altered in Hic-5 knockdown CAFs [95]. Similarly, palladin is an actin-binding protein product of CAFs that is indicated in stromal remodeling. Upregulated in CAFs in four different types of adenocarcinoma, it was hypothesized to impact tumor aggressiveness. Increased palladin expression in CAFs is associated with a poor prognostic outcome in colorectal cancer [41].

CAF interaction with the TME is not limited to secreted factors. CAFs secrete microvesicles as a key facilitator of cell–cell communication and tumor–stroma interaction. CAF-derived microvesicles mediate miRNA transfer to normal fibroblasts, leading to their transformation into CAFs in some instances. Exosomes are a subtype of microvesicles that are secreted by CAFs and contribute to tumorigenesis and metastasis when their contents are horizontally transferred. Chen et al. reported that exosomes released by CAFs promote breast cancer proliferation by transferring miR-500a-5p to cancer cells [96]. The horizontal transfer of miRNA by CAF-derived microvesicles may also contribute to therapy resistance [97]. Sansone et al. demonstrated that microvesicle-mediated miR-221 transfer increased hormonal therapy resistance (HTR) in a luminal breast cancer model [98]. A study by Zhang et al. demonstrated that CAF-derived exosomes transfer miR-522 to tumor cells, triggering ferroptosis inhibition, leading to tumor advancement and lowered susceptibility to chemotherapy [99]. Extracellular vesicles released by ovarian cancer cells can induce fibroblast differentiation into fibroblasts [100].

These findings establish that cell–cell communication is not restricted to growth factor secretion, but microvesicles and exosomes also play an important role through the transfer and exchange of miRNA and other biological molecules.

## 4. CAFs Modulate Immune Cell Recruitment Leading to Suppressed Immune Response

A major impact of CAFs on cancer progression is the communication and modulation of the immune system (Table 2). CAFs and monocytes share significant crosstalk and influence factors such as invasion and prognosis. CAFs are partly responsible for recruiting macrophages to the tissue stroma. As previously discussed, type VI collagen [42] and SDF-1 [72] are factors secreted by CAFs that aid in the recruitment of macrophages. Once recruited, CAFs also play a part in differentiating these macrophages into the M2 phenotype.

Once macrophages are differentiated into M2 macrophages, these cells are involved in pro-tumor activity. They are involved in phagocytosis, immune suppression, angiogenesis, and the alteration of the extracellular matrix. The polarized M2 macrophages continue to communicate with tumor cells, CAFs, and other immune cells to propagate invasion [101].

Experiments performed by Zhang et al. demonstrate that PDAC CAFs induce the differentiation of monocytes into M2 macrophages. Once in the TME, M2 macrophages contributed to pro-tumor effects such as cell growth and invasion [84]. Macrophages can also serve as an origin for CAFs, as reported by Chiu-Tsun Tang et al. [102]. Through Smad3 regulation, macrophages underwent macrophage–myofibroblast transition (MMT), furthering lung cancer progression.

In CRC, CAF-secreted IL-8 facilitated the recruitment of macrophages [70]. CAFs upregulate VCAM-1 expression in colorectal cancer cells, which promotes the adhesion of monocytes. Further, the IL-8/CXCR2 pathway regulates the polarization of monocytes into an M2 phenotype. Antibody-mediated neutralization of IL-8 reduced the chemotaxis of monocytes. Similar results were seen when danirixin, a CXCR2 antagonist, was administered.

Monocyte chemoattractant protein 1 (MCP1), also known as CCL2, secreted by CAFs, promotes HNSCC invasion and migration [73]. MCP1 is also active in recruiting monocytes to cancerous tissue. Blocking MCP1 reduced the recruitment of monocytes to breast tumors [72].

**Table 2:** Cancer-associated fibroblasts recruit pro-tumor immune cells while inhibiting recruitment of anti-tumor immune cells.

CAF Subtype	Factor	Effect	Cancer Type	Reference
eCAF	Macrophages	M2 polarization	GC	[103]
Not determined	Monocytes	Differentiation into macrophages and recruitment to tumors	CRC and breast	[72,73]
Not determined	Mast cells	Mast cell degranulation	GC	[104]
Not determined	Natural killer cells	Reduction in NK cytotoxicity	PDAC	[105]
Not determined	Neutrophils	Neutrophil chemotaxis	HCC	[106]
apCAF	Tregs	T-cell differentiation into Tregs	PDAC	[107]
High-CAF	Helper T cells	Reduced helper T population	HNSCC	[108]
apCAFs	Cytotoxic T cells	Suppression of cytotoxic T cells	PDAC	[12]

CAFs also regulate mast cells. In mouse models of peritoneal tumors, CXCL12 from CAFs was suppressed. This reduced mast cell migration and degranulation and reduced tumor fibrosis [104]. Natural killer (NK) cells, an important immune component responsible for eliminating tumor cells, interact with CAFs as tumors progress. In PDAC, CAFs suppress natural killer cells [105]. Neutrophils involved in the immune response to cancer are also affected by CAFs. In HCC, CAFs recruited neutrophils via chemotaxis and protected them against apoptosis [106]. Protection from apoptosis occurred via activation of the IL6-STAT3 in neutrophils. Neutrophil survival benefits tumor progression, as these neutrophils suppress the T-cell immune response against the cancer.

Stromal fibroblasts communicate with several types of T cells in the cancer environment, further modulating the immune response to cancer. One study involving pancreatic cancer identified apCAFs, a classification of CAFs expressing MHCII molecules. These apCAF directly ligated to CD4 T cells, inducing differentiation into Tregs [107].

In GC, CAFs categorized as high risk with the CAFS-score criteria developed by Mak et al. had a reduced amount of helper T cells [109]. Cytotoxic T cells expressing CD8 levels are often reduced in cancer, leading to a suppressed immune response. In esophageal cancer, cancer cells cultured with CAFs resulted in a reduction of CD8^+^ T cells and a more aggressive cancer. Blocking the IL-6 pathway of signaling in CAFs led to a restoration of CD8^+^ T cells and hindered tumor growth [110]. In single-cell RNA sequencing experiments with gastric cancer, iCAFs chemoattract T cells through the secretion of IL-6 and CXCL12 [103].

## 5. Conclusions

Cancer cells can no longer be studied in isolation. The tumor microenvironment communicates with tumors at every step of progression, and CAFs play a large role in this process. The heterogeneity of CAFs, with both pro-tumor and anti-tumor functions, makes the development of a universal treatment difficult. However, a better understanding of the role of CAFs in specific tumor types will facilitate improved therapeutic approaches. CAFs release a multitude of factors that influence tumor cells, thus facilitating the aggressiveness of the cancer. They modulate the ECM, perfecting the surrounding environment for cancer progression. A modified ECM paves the way for tumor invasion and is an alternate mode of tumor regulation by CAFs. Additionally, CAFs can communicate with immune cells, attenuating or enhancing the normal anti-tumor response. Feedback loops initiated by stromal fibroblasts allow the cancer to snowball into metastasis, making treatment difficult. Understanding the heterogeneity of CAFs is key to unlocking the next step in cancer research and treatment.

## Figures and Tables

**Table 1:** Signaling molecules and growth factors secreted by CAFs into the TME.

Factor	Receptor	Reference
TGF-β1 and TGF-β2	TGF-βR type I and II	[61,62,63,64,65,66,67]
IL-6	IL-6R	[68,69]
IL-8	CXCR1/2	[70,71]
MCP1 (CCL2)	CCR-2	[72,73,74]
HGF (SF)	c-Met	[75,76]
LIF	LIFR	[77,78]
SDF-1 (CXCL12)	CXCR4	[8,79]
CXCL1	CXCR2	[48,80,81]
FGF1, FGF2, and FGF9	FGFR, FGFR2, and FGFR9	[82,83]
CCL-5	CCR1, CCR3, and CCR5	[84]
IL-11	IL-11R	[84]

## Abbreviations

Tumor microenvironment (TME), extracellular matrix (ECM), cancer-associated fibroblasts (CAFs), α-smooth muscle actin (α-SMA), transforming growth factor beta (TGF-β), transforming growth factor beta receptor (TGF-βR), platelet-derived growth factor (PDGF), platelet-derived growth factor (PDGFR), hepatocyte growth factor (HGF), fibroblast growth factor 2 (FGF2), fibroblast growth factor receptor (FGFR), fibroblast-specific protein 1 (FSP1), fibroblast activation protein (FAP), C-X-C motif chemokine 12 (CXCL12), leucine-rich repeat containing 15 (LRRC15), interleukin-6 (IL-6), matrix metalloproteinases (MMPs), tissue inhibitors of metalloproteinases (TIMPs), interleukin 8 (IL-8), interleukin-11 (IL-11), interleukin-11 receptor (IL-11R), monocyte chemoattractant protein-1 (MCP1), C-C chemokine receptor type 1 (CCR-1), C-C chemokine receptor type 2 (CCR-2) C-C chemokine receptor type 3 (CCR-3) C-C motif chemokine ligand 5 (CCL-5), mesenchymal-epithelial transition factor (c-MET), leukemia inhibitory factor (LIF), leukemia inhibitory factor receptor (LIF-R), stromal cell-derived factor 1 (SDF-1), C-X-C motif chemokine 1 (CXCL1), C-X-C chemokine receptor 2 (CXCR4), C-X-C chemokine receptor 4 (CXCR4), lysyl oxidase (LOX), hepatocellular carcinoma (HCC), colorectal cancer (CRC), head and neck squamous cell carcinoma (HNSCC), Sex determining region Y-box 2 (SOX2), vascular cell adhesion molecule (VCAM-1), Hydrogen peroxide-inducible clone 5 (Hic-5) yes-associated protein (YAP), collagen XI α1 (colXIα1), transcriptional coactivator with PDZ-binding motif (TAZ), glycoprotein dickkopf-3 (DKK3), β-aminopropionitrile (BAPN), discoidin domain receptor 2 (DDR2), and Pancreatic ductal adenocarcenoma (PDAC).
